# Modeling Maize Canopy Morphology in Response to Increased Plant Density

**DOI:** 10.3389/fpls.2020.533514

**Published:** 2021-01-15

**Authors:** Liang He, Weiwei Sun, Xiang Chen, Liqi Han, Jincai Li, Yuanshan Ma, Youhong Song

**Affiliations:** ^1^School of Agronomy, Anhui Agricultural University, Hefei, China; ^2^The Queensland Alliance for Agriculture and Food Innovation, The University of Queensland, Brisbane, QLD, Australia

**Keywords:** *Zea mays*, functional-structural plant modeling, interplant competition, crop ideotype, canopy photosynthesis

## Abstract

Increased plant density markedly affects canopy morphophysiological activities and crop productivity. This study aims to model maize canopy final morphology under increased interplant competition by revising a functional–structural plant model, i.e., ADEL-Maize. A 2-year field experiment was conducted at Mengcheng, Anhui Province, China, in 2016 and 2018. A randomized complete block design of five plant densities (PDs), i.e., 4.5, 6, 7.5, 9, and 15 plants m^–2^, with three replications was applied using a hybrid, i.e., Zhengdan 958. Canopy morphology at different PDs was measured with destructive samplings when maize canopy was fully expanded. The relationship of changes of organ morphology in relation to increased plant density was analyzed based on 2016 data. The ADEL-Maize was first calibrated for the hybrid at 4.5 plants m^–2^ and then revised by introducing relationships identified from 2016 data, followed by independent validation with 2018 field data. A heatmap visualization was shown to clearly illustrate the effects of increased plant density on final morphology of laminae, sheaths, and internodes. The logarithmic + linear equations were found to fit changes for the organ size versus increased plant density for phytomers excluding ear position or linear equations for the phytomer at ear position based on 2016 field data. The revision was then further tested independently by having achieved satisfactory agreements between the simulations and observations in canopy size under different PDs with 2018 field data. In conclusion, this study has characterized the relationship between canopy morphology and increased interplant competition for use in the ADEL-Maize and realized the simulations of final size of laminae, sheaths, and internodes, as affected by increased plant density, laying a foundation to test an ideotype for maize withstanding high interplant competition.

## Introduction

Greater crop productivity and grain quality are required to ensure current and future food security due to continuous expansion of a large population and climate change (Ray et al., 2013; Long et al., 2015). Increasing sowing density is a common maize cultivation practice in China as it is in other countries due to limited land for such a large population (Al-Naggar et al., 2015; Liu et al., 2017). Maize production improvement, in particular, has been due to the greater tolerance of increased plant density (PD) (Tokatlidis and Koutroubas, 2004; Ci et al., 2011) by altering canopy architecture with erect leaves at the top and flat leaves at the bottom (Zhu et al., 2012), made available by the efforts of plant breeders (Chen et al., 2016). However, when PD surpasses a certain limit, there will be a significant loss of maize grain yield due to the diminishing photosynthesis, asynchronous anthesis-silking, and ovary abortion (Sanoi, 2000; Tokatlidis and Koutroubas, 2004; Al-Naggar et al., 2015). However, it is harder to push the limit of PD levels further at this stage due to the shortage of sugar supply (Ciampitti and Vyn, 2011). Thus, it is a straightforward strategy to improve canopy photosynthetic capacity during the flowering stage when kernel set is being determined under high interplant competition (Ciampitti and Vyn, 2011; Long et al., 2015; Liu et al., 2017).

The light distribution within crop canopies is dependent upon the features of plant canopy architecture, such as leaf number, leaf size, shape, curvature, and leaf inclination and azimuth, which is highly spatiotemporally variable. Crop architecture with erect leaves at the upper layer of the canopy has been bred, known as a compact canopy type, which allows irradiance to penetrate the canopy, reaching the bottom leaves for a greater entire canopy photosynthetic capacity (Hammer et al., 2002; Zhu et al., 2012). However, the canopy architectural characteristics are far from being well explored due to lack of powerful tools that can test all combinations of architectural and eco-physiological traits (Zhu et al., 2012; Rötter et al., 2015). Functional–structural plant models (FSPMs) have been developed and widely used as a tool in simulating plant architecture and physiological functions under various environments (Drouet and Pagès, 2003; Song et al., 2008; Ma et al., 2018). Thus, the FSPM enables the component organization in the canopy to be determined by virtual trials, by which an ideal canopy architecture, i.e., ideotype (Mock and Pearce, 1975) that optimizes the use of various resource favoring canopy productivity may be achieved. Using a method for digital plant reconstruction based on stereo cameras to capture the architecture of five genetically diverse rice varieties, Burgess et al. (2017) have studied the canopy-level impact on photosynthetic efficiency and found that a plant type with erectophile leaves allows more efficient penetration of light into lower canopy layers, and this, in turn, leads to a greater photosynthetic potential.

However, most of the research has focused on modeling of individual plants (Yan et al., 2004), and it is relatively rare for plant models to consider the impacts of interplant competition with a few attempts (Fournier and Andrieu, 1999; Ma et al., 2007, 2008) due to the complex interaction between organ kinetics and environments (Ford, 2014). Consequently, understanding how canopy responds to increased PD holds great significance in designing optimal plant population density and predicting maize canopy morphology under increased PD. However, existing models do not take the density factor into account. Given the growth and development of plants under high density changed obviously, it is necessary to add the density module into FSPMs. ADEL-Maize has been developed in simulating three-dimensional (3D) canopy development in an optimal environment based on L-system formalism (Fournier and Andrieu, 1999). This model has provided an open framework that allows incorporating new modules in accounting for environmental effects. For example, this model was successfully revised in predicting the impacts of drought stress on maize canopy development by introducing the canopy response to drought stress (Song et al., 2008). Consequently, ADEL-Maize is a suitable platform that can be extended to simulate canopy production under interplant competition.

Within the above context, the objectives of the present study are to (i) calibrate the ADEL-Maize for a local hybrid at 4.5 plants m^–2^; (ii) establish response functions of canopy production to increased plant population densities; and (iii) revise and validate the ADEL-Maize in simulating canopy morphology in response to increased PDs.

## Materials and Methods

### Field Experiments

A 2-year experiment was carried out at Mengcheng Agricultural Sci-Tech Park, Anhui province, China (latitude 33°09′N, Longitude 116°33′E) with a lime concretion black soil type in 2016 and 2018. The data in 2016 were reported in Sher et al. (2018), and the data in 2018 are reported for the first time. Five PD treatments with three replicates designed to induce differing levels of interplant competition were used, and they are 4.5, 6, 7.5, 9, and 15 plants m^–2^ (referred to as PD4.5, PD6, PD7.5, PD9, and PD15, respectively). A maize cultivar, i.e., Zhengdan 958 (referred to as ZD958), planted widely across China was sown in east–west-oriented rows. Each plot was 9 m long and 5 m wide (eight rows). The row space was 0.6 m at each PD, and the plant space was 0.38 m (PD4.5), 0.28 m (PD6), 0.22 m (PD7.5), 0.19 m (PD9), and 0.11 m (PD15). The population from 4 to 7.5 plants m^–2^ is commonly used by local farmers for this genotype. The five adjacent reference plants in a row representing the average growth status at the middle of each plot were tagged with plastic labels for leaves 5 and 10 to identify the phytomer rank in guiding destructive samplings. In order to maintain non-limiting conditions for water and nutrients, soil samples were taken a few days prior to sowing to determine the nutritional content, based on which base nutrients were used at a rate of 150 kg ha^–1^ for N, 112.5 kg ha^–1^ for P_2_O_5_, and 112.5 kg ha^–1^ for K_2_O; an initial irrigation was applied at 900 cm^3^ ha^–1^, and the subsequent irrigation depended on fully irrigated from sowing to completion of canopy production. Maize seeds were manually sown to achieve a designated density on June 12, 2016, and June 10, 2018. The weeds in the field were removed manually, and the insect pests were rigorously controlled by appropriate pesticide.

Measurement of canopy morphology from each replicate, with destructive sampling of plants, was carried out when the canopy was fully expanded. The detailed approach has been described (Song et al., 2015, 2016). The rank of each phytomer in the plant was counted acropetally. In this study, we measured total and fully expanded leaf number, length of lamina and sheath, lamina maximum width, sheath average width, and internode length and diameter for each phytomer during the destructive sampling. Only canopy size at full expansion was used for the analysis. Daily meteorological data including maximum, minimum, and mean temperature and precipitation from June to October in both 2016 and 2018 were obtained from a standard local weather station located nearby the experimental field. The precipitation and average temperature from sowing to anthesis are shown in Supplementary Figure 1. The total rainfall was 170.1 mm in 2016 and 277.8 mm in 2018. It was a bit drier in 2016 compared to 2018. The average temperature was 27.5°C in 2016 and 28.1°C in 2018.

### Modeling of Morphological Changes Versus Increased Plant Density

Lamina length and width, sheath length and width, and internode length and diameter are affected by increased PD (Andrieu et al., 2006; Song et al., 2016). We attempted to establish the relationships that organ final size changes as a function of PD based on a series of levels of PD in this experiment. Thus, the data were analyzed with different functions embedded in Microsoft Excel.

### Revision of ADEL-Maize

#### Calibration of Parameters for a Local Hybrid ZD958

The final canopy size, i.e., lamina length and width, sheath length and width, and internode length and diameter, at different phytomers for ZD958 at the lowest PD, i.e., 4.5 plants m^–2^, was provided as the base value of canopy size. The total leaf number was 18 in both seasons. The lower rank leaves at phytomers 1–5 cannot be considered due to early dropping off.

#### Insertion of Canopy Morphological Response to Increased Plant Density

The module describing the relationships between canopy sizes, i.e., lamina length (LL), lamina maximum width (LW), sheath length (SL), sheath maximum width (SW), internode length (IL), and internode diameter (ID) at different density levels relative to PD4.5 and increased PD relative to 4.5 was added to the ADEL-Maize model, and the 2018 dataset was used to independently assess the performance of the revisions made to ADEL-Maize using data from the 2016 experiment. The revised model was used to predict the final length of leaf and internode for each phytomer.

#### Validation of Model Simulations

To test the effects of simulation, we used a root mean square error (RSME) to compare the simulation and observation. As there are n sites in the comparisons, the index computed from the observed values (*OBSi*) and the simulation values (*SIMi*) is the normalized root mean square error (NRSME) (Loague and Green, 1991). The simulation is considered excellent with NRMSE ≤ 10%, good if 10–20%, fair if 20–30%, poor if >30% (Jamieson et al., 1991).

(1) $$N\,R\,M\,S\,E=\sqrt{\frac{\Sigma_{1}^{n}\,{\left(S\,I\,M_{i}-O\,B\,S_{i}\right)}^{2}}{n}}\times \frac{1}{\bar{O\,B\,S}}\times 100\%$$

where *SIMi* and *OBSi* represent simulated and observed values, respectively, and $\bar{O\,B\,S}$ represents the observed mean value.

Once validated, a visualization of integrative effects of the maize canopy under PD4.5, PD6, PD7.5, PD9, and PD15, respectively, in 2018 was presented visually to show the impacts of increased PD.

## Results

### Canopy Final Size Affected by Increased Plant Density

**FIGURE 1:**
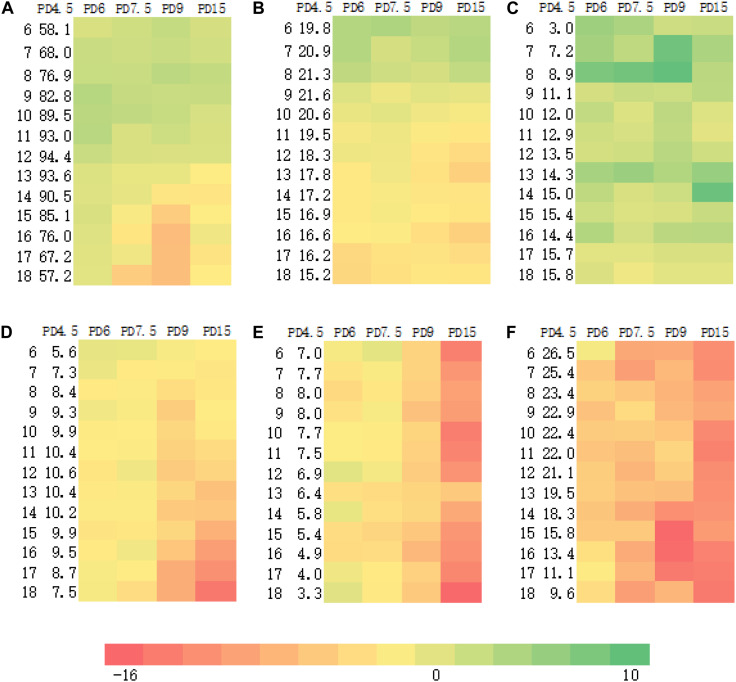
Canopy final size composed of lamina length **(A)**, sheath length **(B)**, internode length **(C)**, lamina width **(D)**, sheath width **(E)**, and internode diameter **(F)** at PD4.5, as affected by increased plant density **(A–F)**, indicated as a visual heatmap in 2016 (horizontal color bar from redness to greenness indicates the changes from –16 to 10%).

The effects of increased PD on canopy final size at anthesis in 2016 are shown in Figure 1. The thermal time was calculated as 992.6°Cd at anthesis in 2016. Lamina length, lamina width, sheath length, sheath width, internode length, and internode diameter at different phytomers from 6 to 18 under 4.5 plants m^–2^ were listed in the second column after phytomer rank (Figure 1). In general, both lamina and sheath widths decreased in response to increased interplant competition while lengths of internodes increased and diameters of internodes were reduced in response to increased PD, consistent with Andrieu et al. (2006) and Song et al. (2016). In this study, the heatmap from column PD6 to PD15 was further made to visually indicate the impacts of increased PD from PD4.5 to PD6, from PD6 to PD7.5, from PD7.5 to PD9, and from PD9 to PD15 on canopy morphology. The more greenness indicates a greater increase due to increased PD, while the more reddish color indicates a greater reduction. Lengths of both laminae and sheaths increased in lower phytomers but decreased in upper phytomers. Consequently, the heatmap is shown quite effective to illustrate the impacts of increased PD with finer details in retrieving the canopy position, organ types, and interplant competition severity.

### Derivation of the Relation Between Organ Size and Increased Plant Density

**FIGURE 2:**
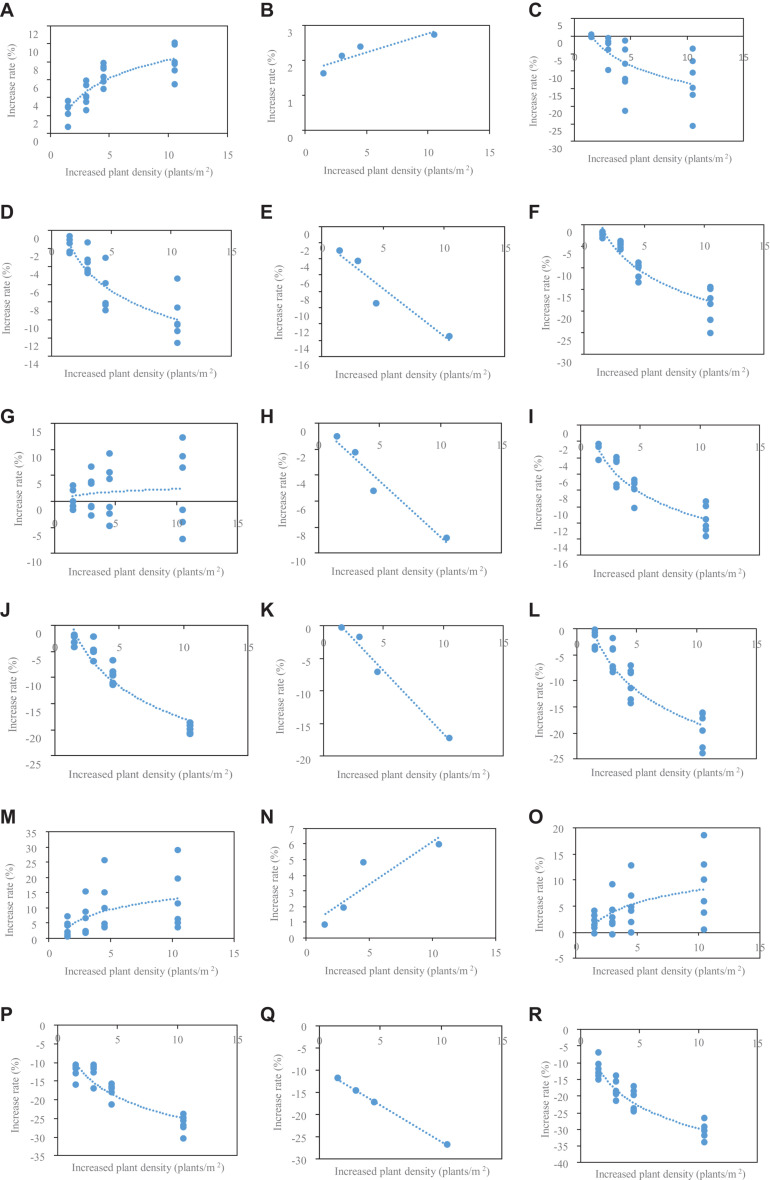
The relationship between the rate of increased LL, lamina length; LW, lamina width; SL, sheath length; SW, sheath width; IL, internode length; and ID, internode diameter at phytomers 6–11 **(A,D,G,J,M,P)**, 12 **(B,E,H,K,N,Q)**, and 13–18 **(C,F,I,L,O,R)** at different densities relative to PD4.5 and increased value of different plant densities relative to 4.5 in 2016.

The relationship between increased rates of organ size at different densities and increased PD relative to PD4.5 in 2016 is shown (Figure 2). The relationship was grouped as phytomers 6–11 (a–p from top to base at first column), phytomer 12 (ear position, b–q from top to base at second column), and phytomer 13–18 (c–r from top to base at third column). For phytomers 6–11 or 13–18, the relationship cannot be fitted by a simple function due to a large variation of the relationship with phytomer position. To account for the effects of phytomer position, we further fitted the relationship by logarithmic + linear equations as Y_(PD_,_PP)_ = A_o_^∗^In(PD) + B_o_^∗^PP + C_o_ for phytomers 6–11 and phytomers 13–18 (A_o_, B_o_, C_o_ denote the parameters, PD denotes plant density, PP denotes phytomer position; o denotes organ type). For phytomer 12, the relationship was found to be nicely fitted by linear equations as Y = D_o_^∗^PD + E_o_ (D_o_, E_o_ denote the parameters, o denotes organ type).

The parameters for A_o_, B_o_, and C_o_ in Table 1 and for D_o_, E_o_ in Table 2 were provided based on the fitting for phytomers 6–11 and 13–18 and phytomer 12. R^2^ is generally high (greater than 80%) except for IL fitting, indicating that the fitting with logarithmic + linear relationships was reasonable in describing the changes of organ size versus increased PD and phytomer position. These equations were introduced into ADEL-Maize, and the parameters were retrieved in the simulations.

**TABLE 1:** The regression of parameters, i.e., A_o_, B_o_, and C_o_ and R^2^ for LL, LW, SL, SW, IL, and ID for two separate groups of phytomers below or beyond ear position in 2016.

**Organ morphology**		**A_o_**	**B_o_**	**C_o_**	**R^2^**
**Type**	**Phytomer position**				
LL	6–11	2.99	0.12	0.32	0.86
	13–18	–7.09	–2.36	39.64	0.88
LW	6–11	–4.34	0.80	7.07	0.94
	13–18	–8.71	0.73	13.93	0.94
SL	6–11	0.73	–2.46	21.63	0.89
	13–18	–4.49	0.61	8.41	0.96
SW	6–11	–8.99	–0.24	4.92	0.95
	13–18	–9.41	0.09	1.55	0.93
IL	6–11	4.94	–2.01	18.61	0.65
	13–18	2.91	–2.06	32.08	0.80
ID	6–11	–7.69	–0.53	–11.36	0.90
	13–18	–9.66	0.07	–8.52	0.93

*LL, leaf length; LW, leaf width; SL, sheath length; SW, sheath width; IL, internode length; ID, internode diameter; A_o_, B_o_, and C_o_ indicate parameters for regression; R^2^ indicates the coefficient of determination.*

**TABLE 2:** The list of parameters, i.e., D_o_ and E_o_ and R^2^ for phytomer 12 at ear position in 2016.

**Organ morphology**		**D_o_**	**E_o_**	**R^2^**
**Type**	**Phytomer position**			
LL	12	0.11	1.70	0.83
LW	12	−1.17	−1.78	0.90
SL	12	−0.86	−0.12	0.95
SW	12	−1.93	2.83	0.98
IL	12	0.54	0.73	0.79
ID	12	−1.63	−9.53	0.99

*LL, leaf length; LW, leaf width; SL, sheath length; SW, sheath width; IL, internode length; ID, internode diameter; o indicates organ types, i.e., lamina, sheath, and internode; D_o_ and E_o_ indicate parameters for regression; R^2^ indicates the coefficient of determination.*

### Characterization of Leaf Angles to Increased Plant Density

The angles between leaf insertion point and vertical stem for three representative leaves under different PDs in 2016 are presented (Supplementary Figure 2). The leaf angle was 18° for the position right below ear (ear position-1), 22° for ear position, and 10° for the position right above the ear (ear position +1) at PD4.5. The angle for the leaf at ear position-1 was significantly reduced when PD reached 6 plants m^–2^ but was not affected further by increased interplant competition. The leaf angle in other positions was not significantly affected by increased PD. Thus, leaf angle in the positions near the ear for the compact type was relatively stable across PDs. The leaf angle detail was used in ADEL-Maize calibration for the experimental hybrid.

### Simulation and Validation

**FIGURE 3:**
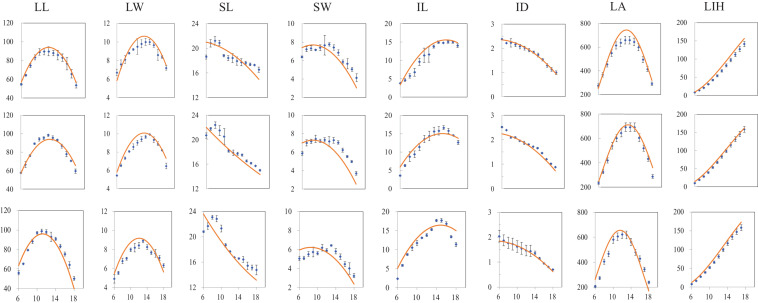
Comparison of simulated (red curves) and measured (scattered dots) values of individual organs, i.e., LL, lamina length; LW, lamina width; SL, sheath length; SW, sheath width; IL, internode length; ID, internode diameter; LA, leaf area; and LIH, leaf inserting height; (vertical coordinate) at different plant densities (PDs) at full canopy expansion, exampled as PD4.5 (top subfigure layer), PD7.5 (middle subfigure layer), and PD15 (bottom subfigure layer) varying with phytomer position (horizontal coordinate) (All subfigures have *x* axis unit as phytomer position; vertical bars indicate standard errors).

The canopy morphology at full expansion for phytomers from 6 to 18 under various PDs in 2018 was simulated with the revised ADEL-Maize in an L-studio platform. The observation data for canopy size at the same time were used to test the simulation under different PDs. The comparison between simulation and observation of canopy morphology including lamina length, maximum width, sheath length, sheath maximum width, internode length and diameter, as well as leaf area (LA), and leaf inserting height (LIH), exampled as PD4.5, PD7.5, and PD15, is shown in Figure 3. It can be seen that the simulations generally agreed well with independent observations from field measurements.

In addition, the calculated value of RMSE in determining the difference between simulation and observation under different PDs in 2018, exampled as PD4.5, PD7.5, and PD15, is shown (Table 3). RMSE values were entirely under 20%, indicating that the simulations for different items were generally acceptable. Of which, RMSE values for SW and IL were relatively large, ranging from 10 to 20%, and the rest fell below 10%, indicating that the simulation for LL, LW, SL, and ID was quite successful, and the simulation for SW and IL should be further improved.

**TABLE 3:** The normalized RMSE values (%) calculated for different organ types under different PDs in 2018, exampled as 4.5, 7.5, and 15 plants m^–2^.

**Organ morphological parameter**	**PD4.5**	**PD7.5**	**PD15.0**
LL	3.89	4.41	7.65
LW	6.51	6.57	9.77
SL	5.84	5.45	8.14
SW	17.74	15.61	18.83
IL	13.69	9.93	14.58
ID	2.77	7.19	5.27
LA	9.85	5.99	12.50
LIH	16.59	6.60	11.58

*PD, plant density; LL, lamina length; LW, lamina maximum width; SL, sheath length; SW, sheath maximum width; IL, internode length; ID, internode diameter, exampled as PD4.5, PD7.5, and PD15. RMSE, root mean square error. The normalized RMSE is expressed as a percentage.*

Therefore, this study suggested that the revised ADEL-Maize to simulate canopy final morphology during flowering in relation to increased PD was acceptable.

### Visualization of Simulations

**FIGURE 4:**
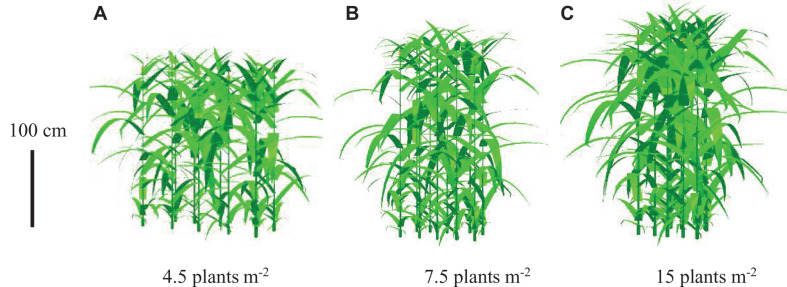
Visualization of maize canopy architecture during flowering under increased plant density, exampled as **(A)** 4.5, **(B)** 7.5, and **(C)** 15 plants m^– 2^ (vertical bar represents 100 cm).

The canopy morphology composed of lamina, sheath, and internode size at different PDs in 2018 was visualized, exampled as PD4.5, PD7.5, and PD15 using the revised ADEL-Maize in an L-studio platform. The effects of increased PD on canopy morphology were visually illustrated as in the images (Figure 4).

## Discussion

### Relations Between Canopy Morphology and Increased Plant Density

To be more profitable, the local farmers tend to cultivate crops in a relatively high PD in achieving greater production under limited farmland. As such, we started from the density at 4.5 plants m^–2^ as the reference, widely adopted for local summer cropping in the south Huang-Huai-Hai region, one of the major maize-producing areas in China, and increased the strength of interplant competition to moderate and even severe level for the rest of the PDs. The complex effects of increased PD on canopy morphology composed of individual organ size can be retrieved by the heatmap (Figure 1). It will help visually demonstrate the impacts of increased interplant competition. Canopy morphology function of increased PD and phytomer position was modeled by fitting equations. The empirical relationships can be used in modeling work. A mechanistic understanding of canopy morphological response to increased interplant competition is essential in the future.

### Model Canopy Architecture as Affected by Increased Plant Density

Maize canopy has the fixed architectural characteristics and a relatively stable competition due to full expansion during the stage bracketing flowering when kernel setting is being determined. The modeling of canopy architecture under various PDs is of benefit to analyze the interaction of 3D organs and environments for coping with interplant competition. Comparing to crop models in accounting for PD (Cazanga et al., 2019), it is more challenging for FSPMs to do so due to the requirement of fine 3D architectural details apart from physiological traits (Drouet and Pagès, 2003; Zhu et al., 2012; Burgess et al., 2017).

ADEL-Maize was employed in simulating 3D canopy production with an open, well-established, and 3D maize modeling platform, allowing implementing specific research aims by introducing additional new modules (Fournier and Andrieu, 1999; Song et al., 2008). The relationships of changes of lamina length and width, sheath length and width, and internode length and diameter versus increased PD have been quantified and introduced to the ADEL-Maize. By inserting relationships into ADEL-Maize, the prediction of canopy morphology response to increased PD was realized and testified by having validated the simulation independently. In this study, we only considered the angles between leaves and the stem for three leaves near the ear from PD effects and validation due to a shortage of data for rest leaves. It is meaningful since the three leaves are most important for grain-filling. This study made an important attempt in modeling the effects of increased PD on canopy morphology; that is, the accumulation of organ dynamics as affected by increased interplant competition. It is noteworthy that these relationships may vary with hybrids, row configuration, etc., thus have to be calibrated in specific cases. The canopy morphology under various PDs received irradiance by the leaf element induced by interplant and intraplant competition. Therefore, a more mechanistic understanding in driving canopy development as a function of irradiance will be necessary in the future (Ford, 2014).

Consequently, the tool for plant functional–structural modeling, i.e., the revised ADEL-Maize will be able to test the impacts of altering canopy architecture including leaf size, shape, angles, which is subjected to various PDs and plant arrangements (Qi et al., 2010; Burgess et al., 2017). This allows studies to achieve a plant ideotype under high interplant competition by adjusting the parameters, and thus canopy photosynthesis can be maximized.

### Implications in Modeling Canopy Photosynthesis as Affected by Increased Plant Density

High PD cropping should overcome the failure of seed setting by supplying sufficient carbohydrates at the developmental stage of bracketing flowering (Sanoi, 2000). In order to enable the revised model for assisted analysis of boosting canopy productivity under high PDs, model light distribution in the canopy and canopy photosynthesis according to irradiance should be done in a further study. In the early stage of FSPM, light distribution can be simulated with a traditional approach, i.e., light extinction with a function of leaf area index (Maddonni and Otegui, 1996; Yan et al., 2004), which may treat the canopy as a big leaf model or a two-leaf model or multilayer model like the way it is treated in crop models. For example, light interception by the individual plants has been simulated with ADEL-Maize (Pommel et al., 2001). The FSPM has a very delicate description of plant architecture, enabling to model light distribution for each structural element precisely. In particular, different models for precise simulation of irradiance in the canopy have been developed (Chelle and Andrieu, 1998, 1999; Cieslak et al., 2008; Wang et al., 2008; Henke and Buck-Sorlin, 2018). For instance, an efficient QuasiMC based on photons tracing was available in simulating canopy light environments rapidly (Cieslak et al., 2008). Thus, the QuasiMC can be employed in the revised ADEL-Maize to model irradiance distribution in the canopy under various PDs.

Once irradiance received by each leaf unit is obtained, a photosynthetic model will be applied to calculate leaf and canopy photosynthesis. Empirical models of leaf photosynthetic light response may be used to estimate canopy photosynthesis (Hammer et al., 2009; review in Wu et al., 2016). Notably, recently, the mechanistic leaf level photosynthetic model is receiving much attention in linking molecular to whole plant level for crop improvement (Wu et al., 2016, 2018). Canopy photosynthesis can be scaled up from integration of leaf level photosynthetic simulation (Wu et al., 2016, 2019).

## Conclusion

To enhance canopy photosynthesis for maize withstanding high interplant competition, modeling canopy morphology and architecture in response to increased PDs is a key focus. The effects of increased PD on lamina length and width, sheath length and width, and internode length and diameter were illustrated with a heatmap visualization based on which the changes with regard to increased PD relative to the PD4.5 could then be further characterized by logarithmic and linear equations for phytomers excluding ear position and linear equations for the phytomer bearing an ear, respectively. ADEL-Maize has been revised by having (i) calibrated the model parameters for the local hybrid, and (ii) inserted the equations identified into the model. The revisions were also successfully validated by achieving satisfactory agreement between simulations and observation from an independent dataset. The visualization of canopy production at different PDs has been completed by visually showing the effect as a whole. To allow the revised model to be used in designing an ideotype to maximize canopy photosynthesis to overcome ear tip abortion due to lack of carbohydrate supply, it is necessary to further incorporate a model of irradiance distribution within the canopy and a biochemical model of C_4_ leaf photosynthesis under various PDs. In summary, this study has made an important step forward in providing a valuable tool that helps define an ideotype for maize cropping under high interplant competition.

## Data Availability Statement

All datasets generated for this study are included in the article/Supplementary Material.

## Author Contributions

LHe did the experiments and modeling analysis and drafted the manuscript. WS contributed the modeling analysis and draft. XC and LHa contributed to modeling analysis. JL and YM contributed to designing the experiments. YS conceived this study and finalized the manuscript.

## Conflict of Interest

The authors declare that the research was conducted in the absence of any commercial or financial relationships that could be construed as a potential conflict of interest.
