# Supplementary material for: Modeling Maize Canopy Morphology in Response to Increased Plant Density
Source: Front Plant Sci. 2021 Jan 15;11:533514. doi: 10.3389/fpls.2020.533514 (PMC7843585; doi:10.3389/fpls.2020.533514)
Supplement: Supplementary file 1 [file Data_Sheet_1.docx]

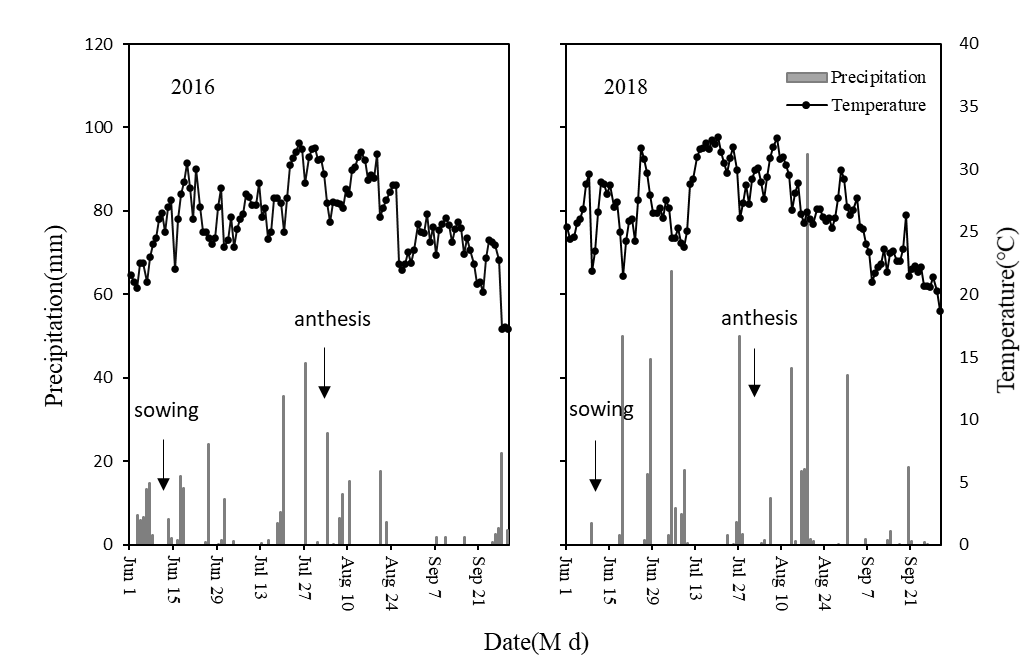


Supplementary Fig. 1. Time course of precipitation (mm) and average temperature (^o^C) from June 1st to September 30th in 2016 and 2018. Arrow indicates the timing of sowing and anthesis.
