# Supplementary material for: Modeling Maize Canopy Morphology in Response to Increased Plant Density
Source: Front Plant Sci. 2021 Jan 15;11:533514. doi: 10.3389/fpls.2020.533514 (PMC7843585; doi:10.3389/fpls.2020.533514)
Supplement: Supplementary file 2 [file Data_Sheet_2.docx]

A

B

C

Supplementary Fig. 2. The angle between leaf lamina (the area connecting the sheath) and vertical stem for leaf at ear position-1 (A), ear position (B) and ear position +1 (C) in 2016 (Vertical bars indicates standard errors and letter a, b indicate the significance at 0.05 level).
